# Supplementary material for: Epidemiology of substance use and mental health disorders among forced migrants displaced from the MENAT region: A systematic review and meta-analysis protocol
Source: PLoS One. 2023 Oct 5;18(10):e0292535. doi: 10.1371/journal.pone.0292535 (PMC10553213; doi:10.1371/journal.pone.0292535)
Supplement: Attachment — Submitted filename: Rebuttal Letter.docx [file pone.0292535.s002.docx]

28th July, 2023

Dear Editorial Board,

We extend our sincere appreciation for the valuable and insightful feedback provided by the reviewers regarding our manuscript titled "**Epidemiology of substance use and mental health disorders among forced migrants displaced from the MENA region: a systematic review protocol**". We are pleased to submit the revised version of our manuscript to PLOS ONE journal.

The reviewers' comments have significantly contributed to the enhancement of our manuscript. Below, we have addressed each comment raised and incorporated the necessary revisions accordingly. Furthermore, we affirm that the present work has not been published in any journal previously, nor is it under consideration for publication elsewhere.

Sincerely,

Maryam Kazemitabar, Ph.D.

Department of Internal Medicine, Yale University

Email: [maryam.kazemitabar@yale.edu](mailto:maryam.kazemitabar@yale.edu)

**Please see our responses to the reviewers’ comments below. My responses to the reviewers' comments are in bold black and when I directly referred to the manuscript contents in bold blue.**

**Reviewer #1**: This protocol outlines a well-designed study and important study on the epidemiology of substance use and mental health disorders among MENA forced migrants. The authors motivate the study well and prose a technically sound study that should achieve their research aims. Notwithstanding these considerable strengths, the submission could be strengthened through several additional considerations:

1. Introduction:

a. It would be useful to add a few lines articulating exactly why the MENA population warrants further study, other than there being a literature gap. After all, if the state of the literature is so poor, then why conduct a systematic literature review and not something more exploratory, such as a scoping study? It would be worth providing a short review of the sizable literature on the mental health needs and experiences of the MENA population and to position this study in relation to other available reviews.

**Response to the reviewer’s comment: Thank you for your helpful comment. I revised it, you can see the changes on pages 3 and 4 lines 77-83: “Studying the epidemiology of SUDs and MHDs among forced migrants from the MENA region is crucial due to their unique challenges and vulnerabilities. There is a substantial body of existing research on the topic of SUDs [12, 13] and especially MHDs [14-18] from the MENA region. However, the availability of systematic reviews that comprehensively collect and analyze this data is limited. Conducting a systematic literature review will help bridge this gap by providing a comprehensive synthesis of the existing research, allowing for a more thorough understanding of the epidemiology of SUDs and MHDs among this population.”**

2. Inclusion/exclusion criteria:

a. The authors’ definition of “forced migrants displaced from the MENA region” could be sharpened. Taken at face value, this wording suggests that the authors will only include studies on people that have been displaced from the region entirely, which would exclude the steep majority, who have been displaced within the region. From the search terms, I don’t believe this is the authors’ intention, but it would be worth stating in the inclusion/exclusion criteria section exactly what they intend. For instance: studies focused on people from the MENA region who have been forcibly displaced by conflict, persecution, and/or natural disaster, including international displacement as well as internal displacement.

**Response to the reviewer’s comment: Thank you for your valuable feedback. We intended to include both internationally and internal displacement, so we included this sentence per your suggestion. Please see page 6, lines 140-142: “Studies focused on people from the MENA region who have been forcibly displaced by conflict, persecution, and/or natural disaster, including international displacement as well as internal displacement will be included.”.**

Relatedly, the authors’ definition of the MENA region is a little fuzzy, since UNOCHA responds to both places producing displacement (e.g., Syria) and places receiving displaced people (e.g., Jordan). Often, for example, Turkey is not included in definitions of the MENA region (for instance, in OHCHR), but UNOCHA includes it because of its Syrian response.

**Response to the reviewer’s comment: Thank you for your insightful comment. We used United Nations Human Resources Office of the High Commissioner (HROHC) classification for included countries in MENA region plus Türkiye (MENAT).**

Some related questions: Is there a minimum/maximum length of time for the length of displacement that the authors will include? For instance, will they include resettled refugees, who are technically no longer displaced? Will they include people who fled a natural disaster for a month, only to return? Will they include returnees more generally?

**Response to the reviewer’s comment: There is no specific length of time for displacement in this study. All refugees resettled or not resettled will be included since even after resettlement they might struggle with SUDs and/or MHDs, we decided to include them all.**

b. Will the authors include studies that include participants from other regions, or only studies that focus exclusively on migrants from the MENA region?

**Response to the reviewer’s comment: Only studies on forced migrants from the MENA region. Expanding the inclusion criteria to encompass forced migrants from other regions would significantly increase the number of extracted articles, making the process of data extraction and synthesis unmanageable. Furthermore, there already exists a substantial body of research focusing on MHDs among forced migrants specifically from the MENA region.**

c. The authors justify the 2013-2023 period with a parenthetical reference to the Arab Spring, but it’s unclear how this fits exactly. The Conventional periodization of the Arab Spring is 2010-2012, so is the goal to include only studies that followed the Arab Spring? Why not also include 2010-2012? As an aside, the introduction section attributes displacement to the Arab Spring protest, rather than to the state crackdowns, coups, and war that followed. I would consider reframing.

**Response to the reviewer’s comment: We aim to include the studies conducted in the last ten years not more to make the process of data extraction and synthesis manageable. The introduction section is reframed per your suggestion. Please see page 3, lines 59-62: “The Arab Spring protest and the subsequent state crackdowns, coups, and wars that followed the initial protests played a significant role in the displacement such that caused more than 3.5 million people to get internally displaced; ever since the numbers have even increased to more than three times.”**

d. Will the authors include qualitative, quantitative studies, and mixed-methods studies, or only quantitative studies?

**Response to the reviewer’s comment: All qualitative, quantitative studies, and mixed-methods studies will be included. We added this information to the methods section. Please see page 6, lines 133-134: “The systematic review will include all qualitative, quantitative studies, and mixed-methods studies focusing on SUDs and/or MHDs among forced migrants displaced from the MENA region.”**

3. Search strategy:

a. Concept 1: Consider adding: Türkiye (official name), Kurdistan, and Near East

b. Concept 2: Depending on your responses to the above, consider adding asylee, resettled, and returnee

c. Concept 3: Consider adding regionally specific substances, such as khat/qat, hookah/narghile/argila/shisha/waterpiped. Consider adding search terms in Arabic

**Response to the reviewer’s comment : Thank you for these suggestions about additional search terms. We have added them all, as well as demonyms for Turkish and Kurdish people, and some additional synonyms/spellings for the regionally specific substances.**

**Regarding the suggestion of adding search terms in Arabic: we considered this approach, but we decided not to implement it. We are confident that we can nevertheless expect to identify relevant Arabic-language papers with the current approach, through three pathways. First, many Arabic-language papers in the databases we are searching have English-language abstracts, through which our search strategy will retrieve them. Second, Arabic-language papers in the databases which we are searching with controlled vocabulary will be retrieved by our use of subject headings and our use of the Ovid multipurpose field (as opposed to the Ovid textword field). Third, papers (in any language) in journals that are not indexed in any of the databases which we searched can still be identified in this project through citation chaining, which we will conduct with the tool Citation Chaser (Haddaway 2022), using the bibliographic database Lens, which includes publication metadata from the database/dataset OpenAlex (**[**https://about.lens.org/release-8-5/**](https://about.lens.org/release-8-5/)**), which is recognized as having particularly good coverage of open access journals based in LMIC countries (Khanna 2022). Via these pathways, we hope to identify relevant papers in Arabic, even if they are from journals not indexed in any of the bibliographic databases we are searching. What’s more, we intend to create a supplemental table of relevant articles in other languages – Persian, French, Turkish, any other language – that we identify during the screening process, for use by other researchers studying this topic in the future.**

**Khanna, S., Ball, J., Alperin, J. P., & Willinsky, J. (2022). Recalibrating the Scope of Scholarly Publishing: A Modest Step in a Vast Decolonization Process. *Quantitative Science Studies*, 1–43.** [**https://doi.org/10.1162/qss_a_00228**](https://doi.org/10.1162/qss_a_00228)

**Haddaway, N. R., Grainger, M. J., & Gray, C. T. (2022). Citationchaser: A tool for transparent and efficient forward and backward citation chasing in systematic searching. *Research Synthesis Methods*, *n/a*(n/a).** [**https://doi.org/10.1002/jrsm.1563**](https://doi.org/10.1002/jrsm.1563)

**Reviewer #2**: Thank you for inviting me to review the protocol “Epidemiology of substance use and mental health disorders among forced migrants 2 displaced from the MENA region: a systematic review protocol”. Overall, I believe that the review topic itself has merit, but this manuscript would benefit from major revisions, and perhaps even the assistance of an editor for help with writing as there is awkward phrasing throughout the paper. I have provided my comments as both a topic expert (migrant health) and as a systematic review methodologist. I hope the authors find my comments useful in preparing their next version.

ABSTRACT

The abstract requires substantial revisions – I suggest that the authors review the PRISMA 2020 for Abstracts Checklist (http://prisma-statement.org/Extensions/Abstracts) to help with the overall reporting and structure. Importantly, the authors should provide an explicit statement of the main objective(s) or question(s) the review addresses in the background and define all acronyms at their first mention (e.g., “SU”, “MH”, “MENA”). The Methods should describe the inclusion criteria and the planned approach for synthesis.

**Response to the reviewer’s comment: Thank you for your constructive feedback. The Abstract is revised and improved now.**

INTRODUCTION

Major:

- Please review the UNHCR statistics and verify if more recent stats are available (2022? 2021?).

**Response to the reviewer’s comment: Data is updated from UNHRC.**

- Many statements are provided without in-text citations (e.g., Lines 51-52; 63-71). Please provide supporting references.

**Response to the reviewer’s comment: References added.**

Minor:

- Lines 77 and 78 are repetitive.

**Response to the reviewer’s comment: Thank you for noticing that. This sentence is modified.**

METHODS

Major:

Given the broad nature of the review questions presented by the authors, and the descriptive planned synthesis and presentation of the results, I strongly recommend the authors to consider whether a scoping review is more appropriate review choice. The authors should justify their choice in their protocol. There are resources available on making this decision, as well as guidance for conduct and reporting:

- Munn, Z., Peters, M. D., Stern, C., Tufanaru, C., McArthur, A., & Aromataris, E. (2018). Systematic review or scoping review? Guidance for authors when choosing between a systematic or scoping review approach. BMC medical research methodology, 18, 1-7. https://bmcmedresmethodol.biomedcentral.com/articles/10.1186/s12874-018-0611-x- JBI MANUAL FOR EVIDENCE SYNTHESIS: SCOPING REVIEWS CHAPTER: <https://jbi.global/scoping-review-network/resources>

- Tricco, AC, Lillie, E, Zarin, W, O'Brien, KK, Colquhoun, H, Levac, D, Moher, D, Peters, MD, Horsley, T, Weeks, L, Hempel, S et al. PRISMA extension for scoping reviews (PRISMA-ScR): checklist and explanation. Ann Intern Med. 2018,169(7):467-473. doi:10.7326/M18-0850. <http://www.prisma-statement.org/Extensions/ScopingReviews>

**Response to the reviewer’s comment: We appreciate the reviewer's thoughtful consideration of the review questions and the recommended resources. We have thoroughly reviewed the suggestions and have reevaluated the appropriateness of the review type for each question.**

**After careful consideration, we agree that the primary question (PQ1) has a broad nature and requires a systematic review approach. We will conduct a comprehensive and methodical search of the literature to identify all relevant studies, followed by a rigorous synthesis and analysis of the findings. A systematic review will allow us to address PQ1 with a high level of evidence and ensure a robust and reliable conclusion.**

**Regarding the secondary questions, SQ1 and SQ2, and primary question 2 (PQ2) we acknowledge that they might benefit from a scoping review approach due to their exploratory and broad scope nature. A scoping review will enable us to map the available evidence, identify key concepts, and provide an overview of the literature without necessarily assessing the quality of individual studies. This approach will help us gain a better understanding of the existing research landscape related to these questions.**

**In our protocol, we will explicitly specify the review type for each question and provide a clear justification for our choice. We will also refer to the recommended resources, such as the Munn et al. (2018) paper on choosing between systematic and scoping reviews and the PRISMA-ScR checklist by Tricco et al. (2018), to ensure that our review follows best practices for conduct and reporting.**

**Please see page 14, lines 240-252: “We aim to investigate a comprehensive range of research questions to gain a thorough understanding of the topic under study. The primary question (PQ1) has been identified as having a broad nature, necessitating a systematic review approach. By following a systematic review methodology, we will conduct a rigorous and exhaustive search of the literature, ensuring the inclusion of all relevant studies and providing a robust synthesis and analysis of the findings.**

**Additionally, we recognize that PQ2, SQ1, and SQ2 might benefit from a scoping review approach due to their exploratory and broad scope nature. Employing a scoping review methodology for these secondary questions will enable us to map the available evidence, identify key concepts, and offer an overview of the literature without necessarily assessing the quality of individual studies. This approach will provide a comprehensive understanding of the existing research landscape related to these questions and facilitate the identification of knowledge gaps and potential areas for future research.”.**

Under “Participants”, the authors should define the term “forced migrants”. For example, will the authors include migrants who cross a border (refugees, asylum seekers) and those who don’t (IDPs?). What types of migrant populations would be excluded (e.g., economic migrants, international students, other?).

**Response to the reviewer’s comment: The definition added per your suggestion.**

If the authors choose to conduct a systematic review, then they must specify interventions and outcomes eligible for inclusion.

**Response to the reviewer’s comment: Thank you for your comment. This systematic review is a systematic review of prevalence and incidence. Including interventions and outcomes is not applicable; it is more applicable for Cochrane-style systematic review of interventions.**

Under “Risk of Bias Assessment” – the authors have selected the Cochrane RoB 2.0 tool to assess intervention studies, however this tool it specifically designed for RCTs and does not adequately assess other non-randomized study designs. The authors should consider ROBINS-I.

**Response to the reviewer’s comment: Thank you for your suggestion. Edited!**

Given that the studies addressing review question #1 are likely to be observational and reporting prevalence, the authors should also consider whether the JBI critical appraisal checklist for prevalence studies would be beneficial. Additional guidance for reporting such evidence is available:

- Munn Z, Moola S, Lisy K, Riitano D, Tufanaru C. Methodological guidance for systematic reviews of observational epidemiological studies reporting prevalence and incidence data. Int J Evid Based Healthc. 2015;13(3):147–153.

**Response to the reviewer’s comment: Thank you for your helpful comment. We will use the JBI critical appraisal checklist for prevalence study that you mentioned. The manuscript’s content edited accordingly.**

Under “Data synthesis” it’s not clear why the authors have not described a synthesis approach for the pooling of prevalence estimates, which would be expected in a systematic review addressing question #1. The charting approach described by the authors would be better suited to a scoping review.

**Response to the reviewer’s comment: The approach to synthesizing prevalence studies is added to the manuscript per your suggestion: “The ‘best evidence synthesis’ will be used to synthesizing prevalence estimates. In implementing a best evidence synthesis approach, this study will prioritize higher-quality studies or those with more robust methodology to guide the synthesis process [29]. Greater weight will be assigned to studies with larger sample sizes, rigorous study designs, or lower risk of bias when summarizing prevalence estimates. By employing this approach, we aim to provide a more reliable and valid synthesis of prevalence data that will be informed by studies of the highest quality and methodological rigor.”**

**29- Slavin, R.E., *Best-Evidence Synthesis: An Alternative to Meta-Analytic and Traditional Reviews.* Educational Researcher, 1986. 15(9): p. 5-11.**

The authors previously described that “studies with high risk of bias will be considered in a sensitivity analysis”, but the methods for this analysis are not described in this section.

**Response to the reviewer’s comment: We appreciate the reviewer's comment. We added a description about methods for sensitivity analysis.**

Given the explicit decision not to pool outcomes, the authors will find it challenging to conduct GRADE assessments, as there may be a large number of single-study estimates. Additionally, the authors have not explicitly defined any outcomes in their eligibility criteria or analysis plan, and so it is unclear what types of outcomes will be assessed using GRADE. Notably, there is currently no GRADE guidance available for the assessment of the certainty of evidence of prevalence data. The authors should describe how they plan to assess the evidence for review question 1.

**Response to the reviewer’s comment: Thank you for your comment. We will use ‘best evidence synthesis’ to synthesizing prevalence estimates as mentioned above.**

Minor:

- Line 97 – it is not necessary to describe PROSPERO and cite Booth et al., your reference should include the citation information necessary to locate your registration.

**Response to the reviewer’s comment: We have included registration ID under the “Design of the systematic review” section. Citation for PROSPERO removed.**

- There is a word missing, Line 99: “The current ___ uses the framework…”

**Response to the reviewer’s comment: Edited! Thank you.**

- The headings “study selection criteria” and “inclusion and exclusion criteria” are redundant – The PRISMSA-P Checklist uses the language “Eligibility criteria” – the authors should review PRISMA-P and ensure that they are adequately reporting all items.

**Response to the reviewer’s comment: Edited! Thank you.**

- Under “screening”, the authors should specify that screening will be done in duplicate

**Response to the reviewer’s comment: Added.**
