# Supplementary material for: Epidemiology of substance use and mental health disorders among forced migrants displaced from the MENAT region: A systematic review and meta-analysis protocol
Source: PLoS One. 2023 Oct 5;18(10):e0292535. doi: 10.1371/journal.pone.0292535 (PMC10553213; doi:10.1371/journal.pone.0292535)
Supplement: Attachment — Submitted filename: Response to Reviewers.docx [file pone.0292535.s004.docx]

September 5, 2023

Dear Editorial Board,

We extend our sincere appreciation for the valuable and insightful feedback provided by the reviewers regarding our manuscript titled "**Epidemiology of substance use and mental health disorders among forced migrants displaced from the MENAT region: a systematic review and meta-analysis protocol**". We are pleased to resubmit the second revised version of our manuscript to PLOS ONE journal.

The reviewers' comments have significantly contributed to the enhancement of our manuscript. Below, we have addressed each comment raised and incorporated the necessary revisions accordingly. Furthermore, we affirm that the present work has not been published in any journal previously, nor is it under consideration for publication elsewhere.

Sincerely,

Maryam Kazemitabar, Ph.D.

Department of Internal Medicine, Yale University

Email: [maryam.kazemitabar@yale.edu](mailto:maryam.kazemitabar@yale.edu)

**Please see our responses to the reviewers’ comments below. My responses to the reviewers' comments are in bold black and the contents in bold blue are those that directly refer to the manuscript.**

Reviewer #1: The authors have thoroughly addressed my comments and produced an improved submission. I have two small remaining notes: First, I find the justification for the systematic review for PQ1 somewhat confusing. In response to Reviewer 2, the authors note that "After careful consideration, we agree that the primary question (PQ1) has a broad nature and requires a systematic review approach." They then write in the body of the text that "The primary question (PQ1) has been identified as having a broad nature, necessitating a systematic review approach." This is further confused by the fact that the authors also justify a *scoping* review for PQ2, SQ1, and SQ2 on the basis of their "broad scope nature." I disagree that "having a broad nature" is what warrants a systematic review, for PQ1, and I believe Reviewer 2 actually suggested a *scoping* review (rather than a systematic review) specifically "given the broad nature of the review questions." This is in keeping with the Munn et al. article on deciding between a systematic and scoping review, where they note that "A key difference between scoping reviews and systematic reviews is that in terms of a review question, a scoping review will have a broader “scope” than traditional systematic reviews..." That said, I agree with the authors that a systematic review is appropriate for PQ1—given the question's focus on synthesizing evidence of prevalence—but I would suggest articulating the justification more clearly. For example, using the same Munn et al. article, the authors could argue that PQ1 meets the systematic review indication for "uncover[ing] the international evidence." My second small comment is that some of the edits have introduced new grammatical errors (e.g. page 3, line 75), so I would recommend a comprehensive copy edit before publishing.

**Response to the reviewer’s comment: Thank you for your helpful comment. It is edited according to your suggestion. Please see page 15 line 276. We also reviewed and edited the whole manuscript for English proficiency.**

Reviewer #2: Thank you for addressing my previous comments and for inviting me to review the revised paper. I appreciate the efforts the authors have made in their protocol. Below, I have a few additional comments that should be addressed prior to publication.

1) The abstract states that you will use the Newcastle-Ottawa Scale and Cochrane RoB, but the methods have been revised to the JBI checklist for prevalence studies and ROBINS-I. This needs to be consistent.

**Response to the reviewer’s comment: Good catch! Thank you for reminding us to edit this in the Abstract.**

2) More details are needed regarding how you plan to analyze the data for PQ1. For example, do you plan to conduct a meta-analysis of prevalence estimates? If yes, will you use a random or fixed effects model? Will these estimates be stratified by MH condition? Will you conduct any assessments for publication bias? You do describe the best evidence synthesis approach to weight studies in your analyses, but it is still necessary to describe the analytic approach. I would consider reviewing the paper by Blackmore et al. below as an example of the type of analyses that could be possible. If the authors choose not to conduct a meta-analysis (or other regression analyses), this choice needs to be justified in the protocol.

Citation: Blackmore R, Boyle JA, Fazel M, Ranasinha S, Gray KM, Fitzgerald G, et al. (2020) The prevalence of mental illness in refugees and asylum seekers: A systematic review and meta-analysis. PLoS Med 17(9): e1003337. <https://doi.org/10.1371/journal.pmed.1003337>
**Response to the reviewer’s comment: Thank you for your valuable suggestion. We considered a meta-analysis for PQ1, focusing on SUDs and MHDs prevalence per your suggestion. Please see pages 14-15, lines 249-271:**

**“Statistical analysis**

**The meta-analysis model will be chosen based on the study design. Given the expected variability among studies, a random-effects model will be considered due to its capacity to account for heterogeneity. If utilizing the ‘meta’ package, the ‘metagen’ function [1] will be applied. Alternatively, for the ‘metafor’ package, the ‘rma’ function [2] will be used. The selected function will be executed by inputting a structured data frame and defining relevant parameters including effect size, standard error, sample size, and study identifiers.**

**To quantify heterogeneity among the studies, I² statistic [3] will be employed to quantify the proportion of total variation in effect estimates that is due to heterogeneity rather than chance. Visual representations of study effect sizes and confidence intervals will be generated through the creation of forest plots, using functions like ‘forest()’ or ‘forest.rma()’. Potential publication bias will be explored and visualized using funnel plots and Egger's test [4] considering a *p*-value < .05. These analyses will be facilitated through dedicated functions available in the chosen R package.**

**Subgroup analyses will be conducted to find potential sources of heterogeneity by categorizing studies based on specific study characteristics. Variables anticipated to introduce heterogeneity will be considered for these analyses. Finally, for each variable, separate subgroups will be created, grouping studies with similar characteristics together. The meta-analysis model will then be applied within each subgroup to calculate the pooled effect estimate and associated confidence intervals. Comparing the effect estimates across different subgroups will allow for the identification of patterns or trends, revealing how each variable may impact the overall results. This process will provide valuable insights into the relative contribution of each characteristic to the observed heterogeneity.”**

**We also updated the abstract to reflect these changes.**

3) The authors have still not addressed the issue with planning to use GRADE methods. Given that the outcomes for PQ2 and SQ1/2 will be scoping review questions and no risk of bias assessments will be complete, no GRADE assessment will be possible for these questions. Additionally, there are no current methods for GRADE assessments for prevalence data (PQ1). My suggestion would be for the authors to acknowledge these limitations and state that no assessment for the overall certainty/strength of the evidence will be performed.

**Response to the reviewer’s comment: Thank you for your feedback. We appreciate your input and have taken it into consideration. As per your suggestion, we have included the following sentence to address the issues you mentioned (page 14, lines 251-253):**

**“Given that the outcomes for PQ2/3 and SQ1/2 will be scoping review questions and no risk of bias assessments will be completed, there will be no evaluation conducted to determine the overall certainty or strength of the evidence.”**

4) In response to reviewers #1's comments, the authors described "papers (in any language) in journals that are not indexed in any of the databases which we searched can still be identified in this project through citation chaining, which we will conduct with the tool Citation Chaser (Haddaway 2022), using the bibliographic database Lens, which includes publication metadata from the database/dataset OpenAlex (<https://about.lens.org/release-8-5/>), which is recognized as having particularly good coverage of open access journals based in LMIC countries (Khanna 2022)."

These details (such as your planned use of the tool citation chaser) should be described in the protocol and referenced appropriately. You should list the software you plan to use for your analysis (e.g., RevMan, STATA, R) and any planned visualizations.

**Response to the reviewer’s comment: Thank you for your insightful comments. We appreciate your attention to detail. We added the following information to the protocol per your suggestion:**

**“Additionally, research articles published in journals not covered by the databases we searched can still be located using citation chaining in this study. We will perform this process using the Citation Chaser tool [5] and the bibliographic database Lens. This database incorporates publication details from the OpenAlex database/dataset, known for its strong coverage of open-access journals in low- and middle-income countries [6]. Citation Chaser is an automated tool that streamlines the process of "citation chasing" in systematic reviews. It uses the Lens.org API to quickly retrieve lists of references from various studies and identify articles that cite a specific study. This eliminates the manual effort traditionally required for cross-referencing and enhances accuracy. The tool can generate lists of both referenced and citing records from sources like PubMed, PubMed Central, CrossRef, Microsoft Academic Graph, and CORE, making systematic review searches more efficient.”**

**27- Haddaway NR, Grainger MJ, Gray CT. Citationchaser: A tool for transparent and efficient forward and backward citation chasing in systematic searching. Research Synthesis Methods. 2022 Jul;13(4):533-45.**

**28- Khanna S, Ball J, Alperin JP, Willinsky J. Recalibrating the scope of scholarly publishing: A modest step in a vast decolonization process. Quantitative Science Studies. 2022 Dec 22:1-9.**

**The statistical analysis and the software and packages used to conduct meta-analysis described in details as explained in response to your previous comment.**

**References**

**1.	Viechtbauer, W., *Conducting meta-analyses in R with the metafor package.* Journal of statistical software, 2010. 36: p. 1-48.**

**2.	Viechtbauer, W. *The R package metafor: Past, present, and future*. in *Research Synthesis 2019 incl. Pre-Conference Symposium Big Data in Psychology, Dubrovnik, Croatia*. 2019. ZPID (Leibniz Institute for Psychology Information).**

**3.	Higgins, J.P., et al., *Measuring inconsistency in meta-analyses.* Bmj, 2003. 327(7414): p. 557-560.**

**4.	Egger, M., et al., *Bias in meta-analysis detected by a simple, graphical test.* Bmj, 1997. 315(7109): p. 629-634.**

**5.	Haddaway, N.R., M.J. Grainger, and C.T. Gray, *Citationchaser: A tool for transparent and efficient forward and backward citation chasing in systematic searching.* Research Synthesis Methods, 2022. 13(4): p. 533-545.**

**6.	Khanna, S., et al., *Recalibrating the scope of scholarly publishing: A modest step in a vast decolonization process.* Quantitative Science Studies, 2022: p. 1-19.**
